# Sotagliflozin Modulation of SIRT1/Nrf2 and PI3K/AKT Signaling Pathway Ameliorates Experimental Liver Fibrosis in Rats

**DOI:** 10.1155/omcl/7684652

**Published:** 2025-12-22

**Authors:** Hossein M. Elbadawy, Mohannad A. Almikhlafi, Mohammed H. Alsubhi, Aya A. Shokry, Hany M. Fayed, Bassim M. S. A. Mohamed, Sherif M. Afifi, Tuba Esatbeyoglu, Reda M. S. Korany, Marawan A. Elbaset

**Affiliations:** ^1^ Department of Pharmacology and Toxicology, College of Pharmacy, Taibah University, 42353, Medina, Saudi Arabia, taibahu.edu.sa; ^2^ Health and Life Research Center, Taibah University, 42353, Madinah, Saudi Arabia, taibahu.edu.sa; ^3^ Department of Pharmacology, Faculty of Veterinary Medicine, Cairo University, Giza, 12211, Egypt, cu.edu.eg; ^4^ Department of Pharmacology, National Research Centre, Medical Research and Clinical Studies Institute, 33 El-Bohouth Street, Dokki, Giza, 12622, Egypt, nrc.sci.eg; ^5^ Department for Life Quality Studies, Rimini Campus, University of Bologna, Corso d’Augusto 237, Rimini, 47921, Italy, unibo.it; ^6^ Department of Molecular Food Chemistry and Food Development, Institute of Food and One Health, Gottfried Wilhelm Leibniz University Hannover, Am Kleinen Felde 30, 30167, Hannover, Germany, uni-hannover.de; ^7^ Department of Pathology, Faculty of Veterinary Medicine, Cairo University, P.O. Box 12211, Giza, Egypt, cu.edu.eg; ^8^ Department of Pathology, Faculty of Veterinary Medicine, Egyptian Chinese University, Ain Shams, 4541312, Egypt; ^9^ Stark Neurosciences Research Institute, Indiana University School of Medicine, Indianapolis, Indiana, USA, indiana.edu; ^10^ Department of Neurology, Indiana University School of Medicine, Indianapolis, Indiana, USA, indiana.edu

**Keywords:** antioxidant, apoptosis, liver fibrosis, Nrf2, oxidative stress, SIRT1, sotagliflozin, thioacetamide

## Abstract

**Background and Purpose:**

Liver fibrosis poses a major global health burden, contributing substantially to morbidity and mortality worldwide. This study aims to assess the potential novel mechanisms behind the anti‐fibrotic effects of sotagliflozin (Sota) in thioacetamide (TAA)‐induced liver fibrosis in rats.

**Experimental Approach:**

To induce liver fibrosis in rats, 100 mg/kg of TAA was injected intraperitoneally triweekly for 6 weeks. Treated groups were orally administered sotagliflozin (10 and 20 mg/kg) for 4 weeks, concurrent with TAA injections.

**Key Results:**

Alongside the histological alterations, the elevation of liver enzymes alanine aminotransferase (ALT) and aspartate aminotransferase (AST), lipid profiles total cholesterol (TC) and triglycerides (TAG), cytokines tumor necrosis factor‐alpha (TNF‐α) and interleukin‐6 (IL‐6), apoptotic markers (caspase‐3 and Bcl2 associated X protein [Bax] BAX), phosphatidylinositol 3‐kinase (PI3K), phosphorylated protein kinase B (p‐AKT), and the lipid peroxidation marker malondialdehyde (MDA) indicated liver dysfunction induced by TAA. Furthermore, indicators of liver fibrosis encompassed reduced levels of albumin, antioxidants; glutathione (GSH), superoxide dismutase (SOD), heme oxygenase‐1 (HO‐1), and nuclear factor erythroid 2‐related factor 2 (Nrf2), antiapoptotic protein B‐cell lymphoma‐2 (BCL2), sirtuin‐1 (SIRT1) expression, and histopathological alterations.

**Conclusion and Implications:**

This study demonstrated that daily oral treatment with sotagliflozin markedly upregulated antioxidant markers such as SIRT1 and Nrf2, attenuated TNF‐α, and reduced apoptotic and fibrogenic markers, thereby protecting against TAA‐induced liver fibrosis. This may have occurred through the augmentation of SIRT1/Nrf2 expression, the inhibition of PI3K/AKT, resulting in the suppression of apoptosis and inflammation.

## 1. Introduction

The incidence of hepatic fibrogenesis is rising worldwide at an alarming pace, with mortality reaching ~2 million deaths annually. Hepatic fibrosis arises from chronic liver diseases, including viral and parasitic infections, hepatotoxins, and environmental insults [1]. Pathologically, it is characterized by persistent inflammation, activation of hepatic stellate cells (HSCs), and excessive extracellular matrix (ECM) deposition [2, 3]. Progressive fibrosis can lead to portal hypertension, bleeding, hepatic encephalopathy, loss of liver function, and hepatocellular carcinoma (HCC) [4–6].

Sirtuin‐1 (SIRT1) is a well‐defined member of the mammalian sirtuin family, comprising seven members: SIRT1 through SIRT7. It operates as a NAD (+)‐dependent deacetylase and is purportedly crucial in modulating mitochondrial biogenesis, circadian rhythm, and glucose and lipid homeostasis. SIRT1 plays a pivotal role in mitigating oxidative stress and inflammation by regulating transcription factors, such as nuclear factor‐erythroid 2‐related factor 2 (Nrf2) [7, 8].

Nrf2 prevents oxidative damage to tissues and cells. Nrf2 modulates the antioxidant defense system by activating the cytoprotective enzyme heme oxygenase‐1 (HO‐1), which reduces inflammation and oxidative stress [9, 10]. Recent studies have demonstrated that the SIRT1/Nrf2 pathway is involved in various in vivo liver fibrosis models [11–13]. Previous research has shown that Nrf2 activation reduces hepatic fibrogenesis [13–15]. SIRT1 regulates the expression of multiple downstream genes by controlling transcription factors, such as Nrf2 [16], which modulates the transcription of antioxidant enzymes to affect cellular oxidative stress and has a well‐documented role in liver fibrosis [16, 17].

The SIRT1/Nrf2 pathway has been implicated in the management of oxidative stress in liver fibrosis; however, improvements in targeting metabolic pathways have also focused on glucose regulation, particularly through sodium‐glucose cotransporters (SGLTs). There are two types of SGLT: SGLT1 and SGLT2. A novel affinity‐purified antibody revealed that the human kidney expresses SGLT2, whereas the small intestine, liver, heart, and lung express SGLT1 [18]. SGLT agonists were found to increase inflammatory and oxidative stress properties when endothelial cells were exposed to angiotensin II, resulting in an increase in reactive oxygen species (ROS) content and several proinflammatory cytokines [19]. Sotagliflozin is an oral hypoglycemic agent that is used in patients with type 1 diabetes mellitus. Sotagliflozin induces its hypoglycemic effect through dual inhibition of SGLT 1 and 2; however, sotagliflozin is 20‐fold more selective to SGLT2 than SGLT1 [18]. Sotagliflozin has been proposed as a potential therapy for cystic fibrosis (Xiubin [20]) and cardiac pressure overload [21]. Still, the exact function of sotagliflozin in liver fibrosis remains unknown [22]. It was, therefore, hypothesized that sotagliflozin may have hepatoprotective effects through the modulation of the Sirt1/Nrf2 and PI3K/AKT pathways, causing a decline in oxidative stress, inflammation, and apoptosis in hepatic fibrosis.

## 2. Materials and Methods

### 2.1. Drugs and Chemicals

Sigma–Aldrich was the supplier of thioacetamide (TAA) (Merck, USA; 163678). Sotagliflozin was purchased from Cayman Chemical (USA; 19141). The highest available analytical grade of substances was utilized.

### 2.2. Experimental Animals and Grouping/Experiment Protocol

We used adult male Wistar rats from the National Research Centre’s (NRC, Egypt) animal house. Six to 8‐week‐old rats weighing 180–220 g were kept in a room at 25°C with a 12 h light/dark interval. Experimental animals received care that complied with national and international ethical standards. The Institutional Animal Care and Use Committee at Cairo University granted approval for all animal studies (Vet CU110520251165).

Twenty‐four rats were maintained for 1 week to acclimatize; subsequently, six rats were randomly allocated to each of the four experimental groups. Group 1 was assigned as the negative control group, where rats were injected twice weekly intraperitoneally (IP) with sterile saline (0.9% NaCl) solution, which was filter sterilized prior to injection. To develop liver fibrosis, rats in Group 2, designated as the “TAA group”, were injected IP with 100 mg/kg body weight. Rats were administered TAA thrice weekly for 6 weeks [23, 24]. Rats in Groups 3 and 4 were administered two daily oral doses of sotagliflozin (10, 20 mg/kg) [25], starting 15 days after the TAA injections and continuing until the end of the experiment.

### 2.3. Preparation of Blood and Tissues

Upon completion of the study period, rats were anesthetized with ketamine (50 mg/kg) and xylazine (25 mg/kg), and blood samples were extracted from the tail vein. They were then sacrificed via cervical dislocation. Samples of serum were frozen at −20°C until used for biochemical analysis. After blood was drawn, rats were euthanized while under anesthesia. The liver tissues were then directly extracted and rinsed in ice‐cold saline before being divided into portions. A portion of each weighted rat’s liver was kept at −80°C for molecular analyses. For immunohistochemistry and histology, a separate part was maintained in 10% buffered neutral formalin.

### 2.4. Measurement of Liver Injury Indicators

Colorimetric techniques were used to measure albumin levels (GenWay, USA; Cat. Number: GB0032) as well as total cholesterol (TC) purchased from Cayman (Cayman, USA, Cat. Number: 10007640) and triglycerides (TAG) purchased from Cayman (Cayman, USA, Cat. Number: 10010303). Colorimetric kits for liver enzymes: “aspartate aminotransferase (AST) and alanine aminotransferase (ALT)” were acquired from “Biomatik, USA; Catalog Numbers: EKE62019 and EKU02211, respectively”. Colorimetric quantifications were performed according to the manufacturer’s instructions for each kit.

### 2.5. Measurement of Lipid Peroxidation and Antioxidant Status

To evaluate oxidative stress and antioxidant defense, “glutathione (GSH) content, malondialdehyde (MDA), and superoxide dismutase (SOD)” levels were measured in liver tissue samples using specific colorimetric assays (BioVision, Milpitas, CA, USA; Cat. Numbers: K464‐100, K739‐100, and K335‐100, respectively). Quantification of HO‐1 levels was performed calorimetrically using an HO‐1 Quantification Kit (BioVision, Milpitas, CA, USA; Cat. Number: E4525‐100). Colorimetric assays were completed in accordance with the manufacturer’s instructions.

### 2.6. Measurement of Inflammatory Cytokines and Apoptotic/Antiapoptotic Markers

ELISA kits obtained from Sunlong Biotech, China (Cat. Numbers: SL0722Ra and SL0411Ra) were used to measure the serum levels of TNF‐α and IL‐6. ELISA assay kits (Sunlong Biotech, China; Cat. Numbers: SL1254Ra and SL0985Ra, respectively) were used to detect SIRT1 and Nrf2, while “MyBioSource (San Diego, California, United States)” provided an ELISA kit (Cat. Number: MBS 702819) to measure PI3K following the guidelines provided by the manufacturer. To assess apoptosis and cell survival, ELISA kits were obtained from Cloud‐Clone Corp., TX, USA (Cat. Number: SEB343Ra and Cat. Number: SEA778Ra, respectively) were used according to the manufacturers’ protocols for each kit. Briefly, samples were applied to ELISA plates pre‐coated with a capture antibody, and unbound components were removed using a two‐step washing process, followed by the addition of the secondary antibody. After incubation and washing, a substrate solution was added, and absorbance was measured at 450 nm using “a microplate reader (ELx 800 absorbance microplate reader; Bio‐Tek Instruments Inc., Winooski, VT, USA)”. Additionally, measurement of the apoptotic marker caspase‐3 was performed via a colorimetric assay kit (Sunlong Biotech Co., Zhejiang, China; Cat. Number: SL0152Ra), following the manufacturer’s instructions for each assay kit.

### 2.7. Gene Expression of Nrf2 and SIRT1 in the Hepatic Homogenate (qRT‐PCR)

The Tissue Ruptor II “(Qiagen, Hilden, Germany)” was used to homogenize the 30 mg of liver tissues that were removed from each rat and immersed in the lysis solution. The lysate was centrifuged for 20 min at 4000 rpm, and supernatants were collected for RNA extraction. QIAamp RNeasy Mini Kit “(Qiagen, Germany; Cat. Number: 74104)” was used to extract and purify RNA from tissue samples. To put it briefly, samples were added to 600 µL of RLT buffer that included 10 μL/mL of β‐mercaptoethanol. After the lysate was cleared, one volume of 70% ethanol was added, and the washing procedures were finished, followed by a DNAse digestion step to eliminate genomic DNA according to the specified protocol, followed by an elution step using RNAse‐free water. In order to do reverse transcription, “the QuantiTect Reverse Transcription Kit (Cat. Number: 205310)” was acquired from “Qiagen in Hilden, Germany”. The procedure involved adding 1 µL of reverse transcriptase enzyme to “4 µL of RT buffer, 1 µL of RT primer mix, and 1 µL of dNTP mix” in a reaction volume of 20 µL. Following the addition of 1 µg of RNA to each reaction, the reaction mixture was incubated for “15 min at 42°C, followed by 3 min incubation at 95°C”.

Quantitative Real‐time PCR (qRT‐PCR) requires the following components: “0.5 µL of each primer at a concentration of 20 p mol, 8.25 µL of water, 3 µL of RNA template, 12.5 µL of the 2x QuantiTect SYBR Green PCR Master Mix (Qiagen, Germany), and 0.25 µL of RevertAid Reverse Transcriptase (200 U/µL) (Thermo Fisher, USA)”. A Stratagene MX3005P real‐time qRT‐PCR device “(ThermoFisher, USA)” was used to conduct the reaction. The primers used are indicated in Table 1 and were provided by Metabion (Germany). To estimate the relevant gene expression quantification, the delta–delta CT (*Δ*ΔCt) as *Δ*ΔCt = *Δ*Ct reference –*Δ*Ct target, and then calculating 2^–*Δ*ΔCt^ as per the Yuan et al. [29] method.

**Table 1:** The target genes and primer sequences for SYBR green qRT‐PCR.

Gene	Forward and reverse	Sequence (5′‐3′)
Nrf2 [26]	F	“CACATCCAGACAGACACCAGT”
	R	“CTACAAATGGGAATGTCTCTGC”
SIRT‐1 [27]	F	“CAC‐CAG‐AAA‐GAA‐CTT‐CAC‐CAC‐CAG”
	R	“ACC‐ATC‐AAG‐CCG‐CCT‐ACT‐AAT‐CTG”
*ß*‐Actin [28]	F	“TCCTCCTGAGCGCAAGTACTCT”
	R	“GCTCAGTAACAGTCCGCCTAGAA”

### 2.8. Histopathology

Each experimental group’s liver samples were removed, cleaned, dehydrated, fixed in 10% neutral buffered formalin, and then embedded in paraffin. Hematoxylin and Eosin staining were used after the paraffin‐embedded blocks were sectioned at a thickness of 5 µm [30] for the investigation of histopathology.

### 2.9. Scores for Histopathological Lesions

Changes in the liver were recorded and categorized as no change (0), mild (1), moderate (2), and severe (3). The modifications received the following grade: Mild changes are less than 30%, moderate changes are between 30% and 50%, and severe changes are greater than 50% [31].

### 2.10. Immunohistochemistry

Sections of liver tissue were deparaffinized in “xylene and then rehydrated in different alcohol grades” [32]. To achieve antigen retrieval, sections were pretreated with a pH 6 citrate buffer for 20 min. Tissue sections were treated with “rabbit polyclonal AKT1 phospho S473 antibody (ab8932; 1:100 dilution rate, Abcam, Cambridge, UK)” and “rabbit monoclonal anti‐Nrf2 antibody (ab62352; 1:100 dilution rate, Abcam, Cambridge, UK)” for 2 h in a humidified room. As a chromogen, the sections were incubated with goat anti‐rabbit IgG H&L (HRP) (ab205718; Abcam, Cambridge, UK) using “3,3′‐diaminobenzidine tetrahydrochloride (DAB, Sigma)”. The slides were mounted using DPX and counterstained with hematoxylin. PBS was used in place of primary antibodies to make the negative control slides.

### 2.11. Statistics

Statistics were done according to Elbaset et al. [33] “Values were assured for normality using the Shapiro test. The outcomes are represented as means ± SD Data were processed by one‐way analysis of variance followed by the Tukey–Tukey‐Kramer post hoc test. GraphPad Prism software (version 10, California, USA) was used to conduct the statistical analysis and create the figures. The significance level was set to *p* < 0.05 for all statistical tests”.

## 3. Results

### 3.1. Impact of Sotagliflozin on Liver Injury Markers in TAA‐Induced Liver Fibrosis

TAA administration markedly elevated serum “ALT and AST” by 454.6% and 378.9%, respectively, while albumin decreased by 63% compared to the control. Treatment with sotagliflozin (10 mg/kg) declined “ALT and AST” by 40.7% and 38.6%, respectively, while the higher dose (20 mg/kg) showed greater improvement, reducing them by 65.1% and 68%, respectively, from TAA values. Albumin levels were improved by 80.1% and 120.3% with low and high doses of sotagliflozin, respectively. The 20 mg/kg dose showed superior effects and nearly normalized liver function parameters, reaching 81.5% of control albumin levels (Figure 1A–C).

Figure 1Assessment of Sotagliflozin’s impact on liver function: ALT, AST, and albumin in TAA intoxicated rats. (A) Serum ALT (U/L), (B) Serum AST (U/L), and (C) Serum albumin (mg/dL). Data are expressed as means ± SD. Significant difference is considered at *p* < 0.05. ∗ vs. in corresponding pairwise at *p* < 0.05, ∗∗ vs. in corresponding pairwise at *p* < 0.01, ∗∗∗ vs. corresponding pairwise at *p* < 0.001. TAA: thioacetamide; Sota: sotagliflozin; ALT: alanine aminotransferase; AST: aspartate aminotransferase.

**Figure:**
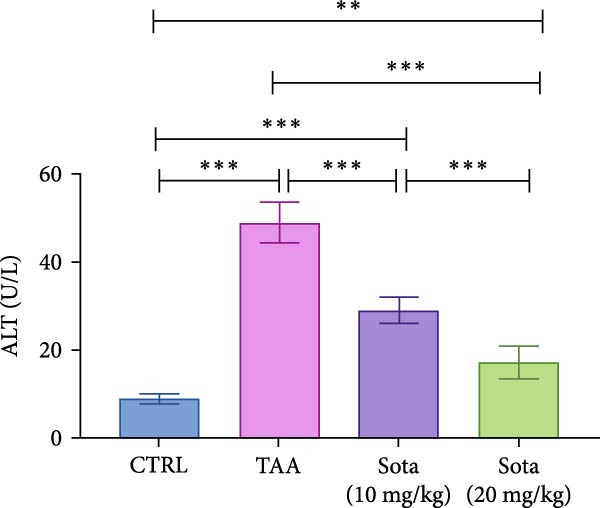
(A)

**Figure:**
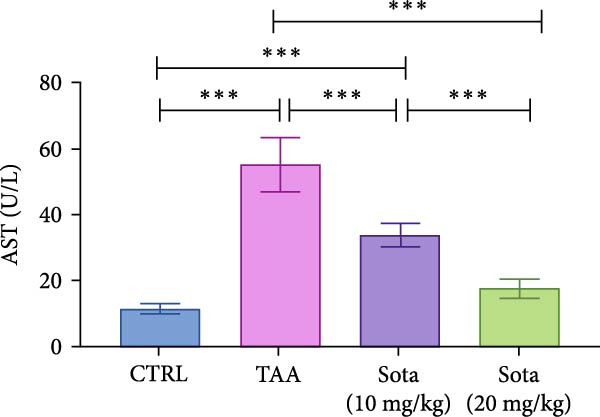
(B)

**Figure:**
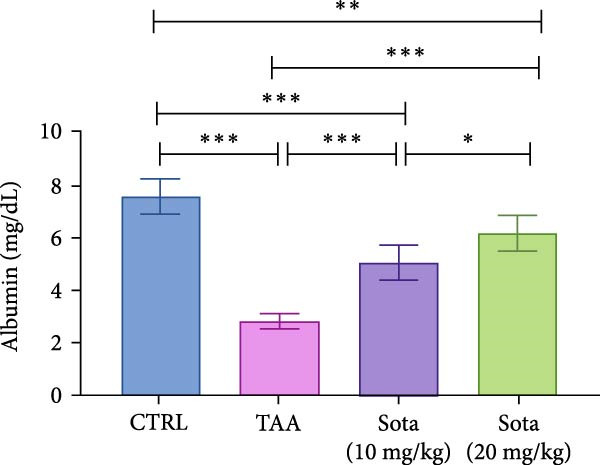
(C)

### 3.2. Impact of Sotagliflozin on Oxidative Stress Markers in TAA‐Induced Liver Fibrosis

TAA induced severe oxidative stress, decreasing GSH and SOD by 86.5% and 73.5%, respectively, while elevating MDA by 227.6% in contrast to the control. Sotagliflozin treatment dose‐dependently improved these markers, with the 20 mg/kg dose showing superior effects. GSH increased by 520.6%, SOD by 208.4%, and MDA decreased by 63.9% compared to the TAA group. The high dose effectively normalized these parameters, with GSH and SOD reaching 83.9% and 81.8% of the control group values, respectively (Figure 2A–C).

Figure 2Sotagliflozin’s effect on oxidative stress markers: GSH, MDA, and SOD in TAA‐intoxicated rats. (A) GSH (nmol/mg protein), (B) MDA (µM/mg protein), and (C) SOD (U/mg protein) activity. Data are expressed as means ± SD. Significant difference is considered at *p* < 0.05. ∗ vs. in corresponding pairwise at *p* < 0.05, ∗∗ vs. in corresponding pairwise at *p* < 0.01, ∗∗∗ vs. corresponding pairwise at *p* < 0.001. TAA: thioacetamide; Sota: sotagliflozin; GSH: glutathione; MDA: malondialdehyde; SOD: superoxide dismutase.

**Figure:**
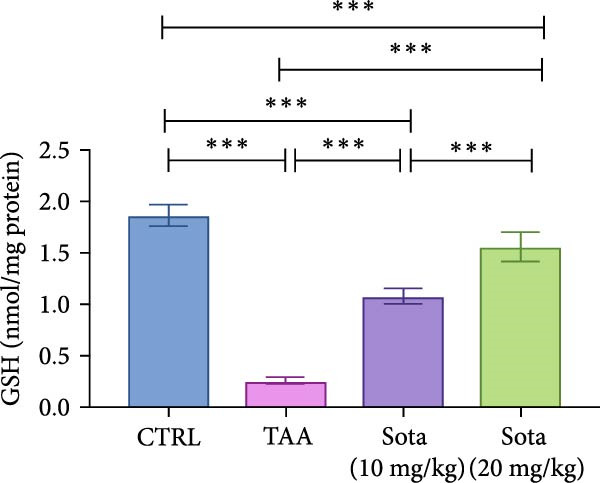
(A)

**Figure:**
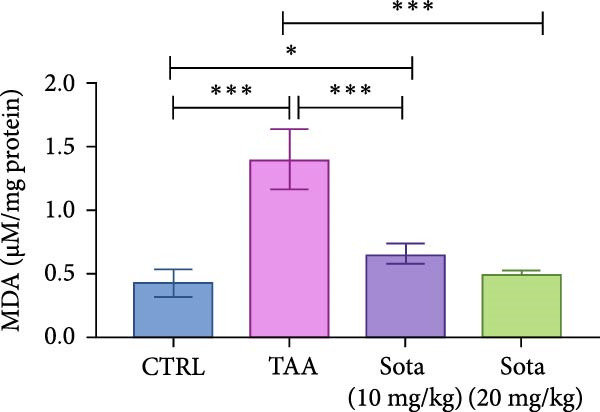
(B)

**Figure:**
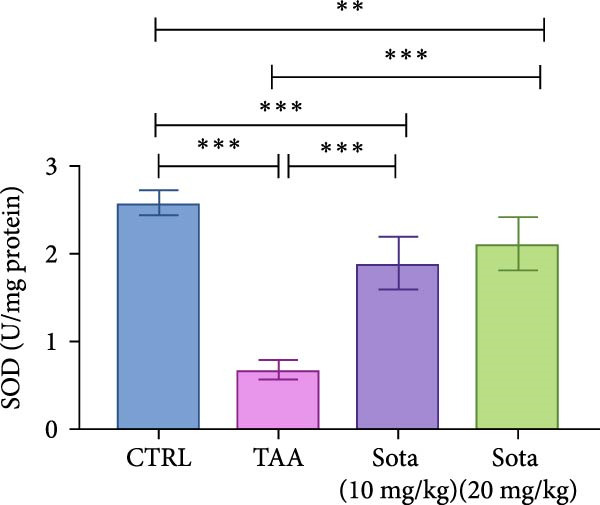
(C)

### 3.3. Impact of Sotagliflozin on Lipid Profile in TAA‐Induced Liver Fibrosis

TAA significantly elevated both TAG and TC by 169.6% and 204.4%, respectively, compared to the control. Sotagliflozin treatment showed dose‐dependent improvement, with the 20 mg/kg dose reducing TAG by 57.3% and TC by 61.5% as opposed to the TAA group. The high dose nearly normalized the lipid profile, bringing levels to 115.2% and 117.1% of control values, respectively (Figure 3A, B).

Figure 3Assessment of Sotagliflozin’s effect on lipid profile: serum triglycerides and total cholesterol in TAA‐intoxicated rats. (A) Serum triglycerides (mg/dL). (B) Serum total cholesterol (mg/dL). Data are expressed as means ± SD. Significant difference is considered at *p* < 0.05. ∗ vs. in corresponding pairwise at *p* < 0.05, ∗∗ vs. in corresponding pairwise at *p* < 0.01, ∗∗∗ vs. corresponding pairwise at *p* < 0.001. TAA: thioacetamide; Sota: sotagliflozin.

**Figure:**
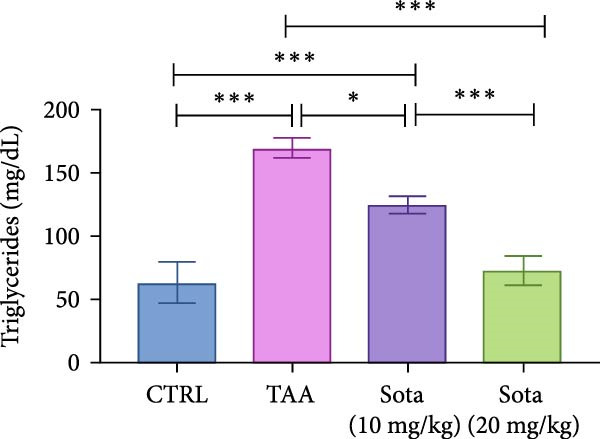
(A)

**Figure:**
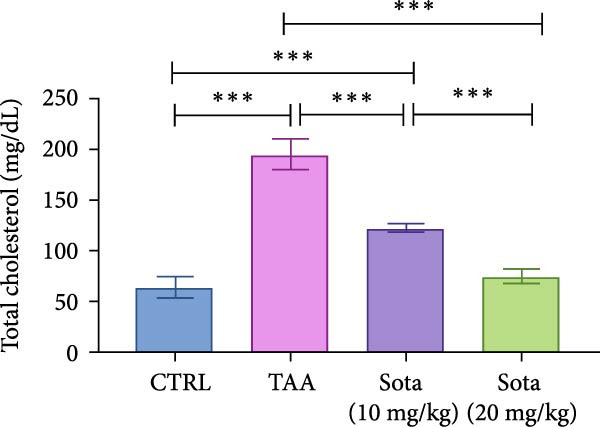
(B)

### 3.4. Effect of Sotagliflozin on Apoptotic Markers in TAA‐Induced Liver Fibrosis

TAA decreased antiapoptotic BCL2 by 69.8% while increasing proapoptotic “BAX and Caspase‐3” by 284% and 347.6%, respectively, as opposed to the control. Sotagliflozin treatment showed dose‐dependent effects, with a 20 mg/kg dose increasing BCL2 by 201.3% and decreasing “BAX and Caspase‐3” by 65.2% and 70.3%, respectively, contrasting with the TAA group. The higher dose nearly normalized these parameters, with BCL2 reaching 91.1% of control values (Figure 4A–C).

Figure 4Impact of Sotagliflozin on apoptosis markers in TAA intoxicated rats. (A) BCL2 (pg/mg protein), (B) BAX (ng/mg protein), (C) Caspase‐3 (ng/mg protein). Data are expressed as means ± SD. Significant difference is considered at *p* < 0.05. ∗ vs. in corresponding pairwise at *p* < 0.05, ∗∗ vs. in corresponding pairwise at *p* < 0.01, ∗∗∗ vs. corresponding pairwise at *p* < 0.001. TAA: thioacetamide; Sota: sotagliflozin; BCL2: B‐cell lymphoma 2; BAX: Bcl2 associated X protein.

**Figure:**
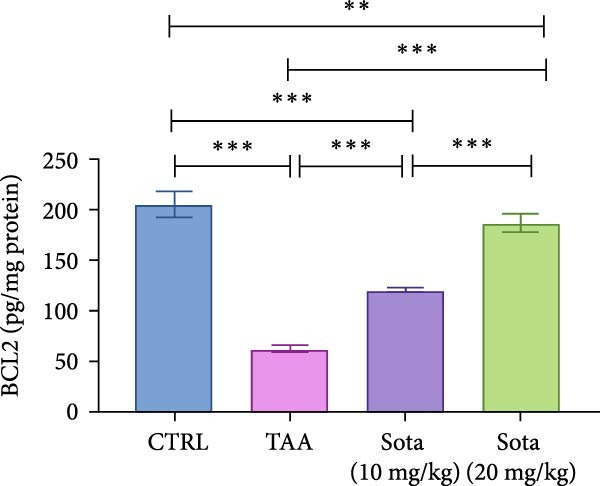
(A)

**Figure:**
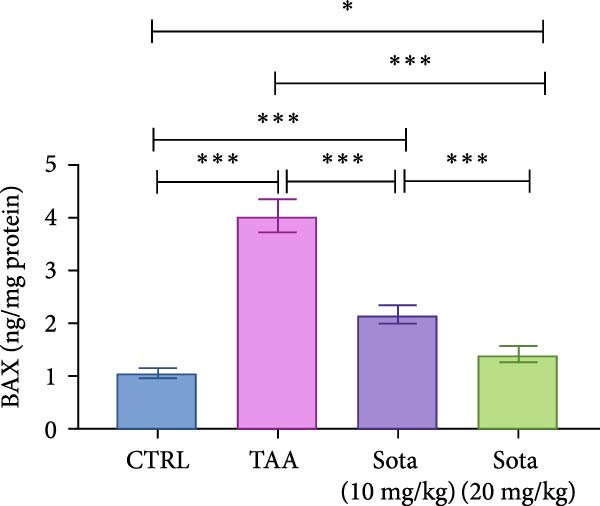
(B)

**Figure:**
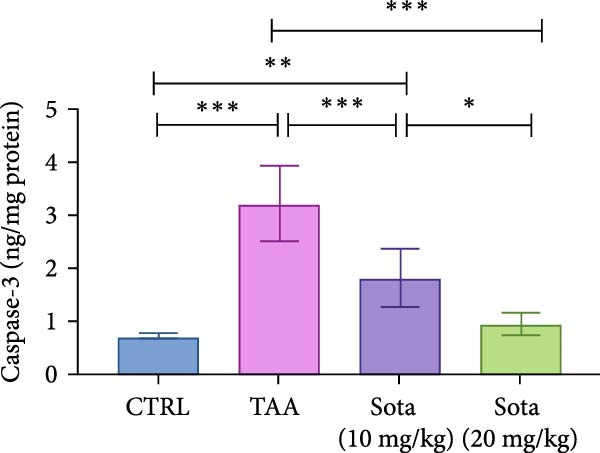
(C)

### 3.5. Impact of Sotagliflozin on Inflammatory Markers in TAA‐Induced Liver Fibrosis

TAA significantly elevated inflammatory markers IL‐6 and TNF‐alpha by 312.9% and 291.1%, respectively, compared to the control. Both doses of sotagliflozin effectively reduced these markers, with the 20 mg/kg dose showing superior effects, reducing IL‐6 by 69.2% and TNF‐alpha by 69.8% compared to the TAA group. The high dose nearly normalized these parameters to 127.2% and 117.9% of the control group values, respectively (Figure 5A, B).

Figure 5Sotagliflozin’s effect on inflammatory mediators: IL‐6, TNF‐α in TAA intoxicated rats. (A) IL‐6 (pg/mg protein). (B) TNF‐α (pg/mg protein). Data are expressed as means ± SD. Significant difference is considered at *p* < 0.05. ∗ vs. in corresponding pairwise at *p* < 0.05, ∗∗ vs. in corresponding pairwise at *p* < 0.01, ∗∗∗ vs. corresponding pairwise at *p* < 0.001. TAA: thioacetamide; Sota: sotagliflozin; IL‐6: interleukin‐6; TNF‐α: tumor necrosis factor‐alpha.

**Figure:**
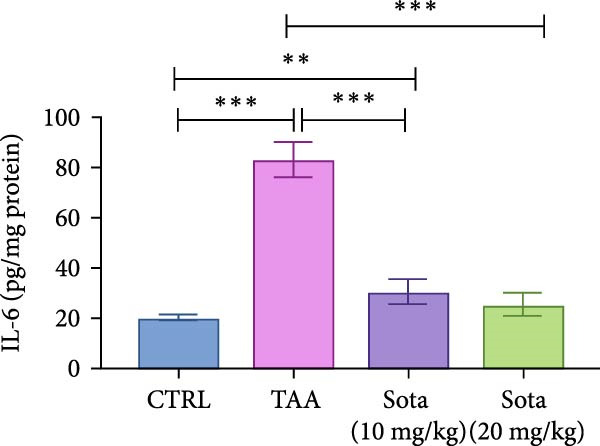
(A)

**Figure:**
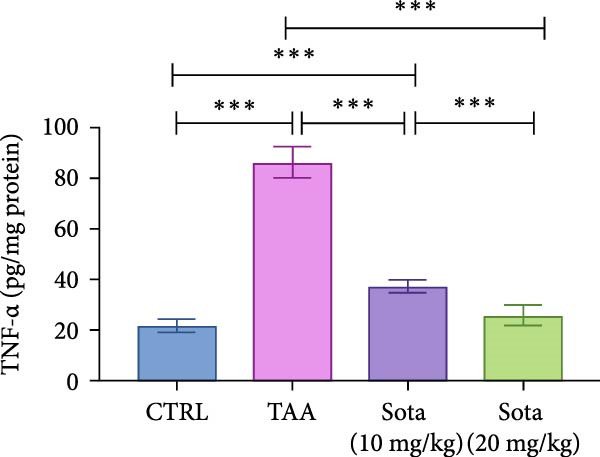
(B)

### 3.6. Effect of Sotagliflozin on PI3K and HO‐1 in TAA‐Induced Liver Fibrosis

TAA increased PI3K by 422.3% while decreasing HO‐1 by 73.9% compared to the control group. Sotagliflozin treatment showed dose‐dependent effects, with the 20 mg/kg dose decreasing PI3K by 78% and increasing HO‐1 by 219.8% compared to the TAA group. The high dose effectively normalized these parameters to 114.9% and 83.4% of the control group values, respectively (Figure 6A, B).

Figure 6Sotagliflozin’s effect on PI3K and HO‐1 in TAA intoxicated rats. (A) “PI3K (ng/mg protein)”. (B) “HO‐1 (ng/mg protein)”. Data are expressed as means ± SD. Significant difference is considered at *p* < 0.05. ∗ vs. in corresponding pairwise at *p* < 0.05, ∗∗ vs. in corresponding pairwise at *p* < 0.01, ∗∗∗ vs. corresponding pairwise at *p* < 0.001. TAA: thioacetamide; Sota: sotagliflozin; PI3K: phosphatidylinositol 3‐kinase; HO‐1: heme oxygenase‐1.

**Figure:**
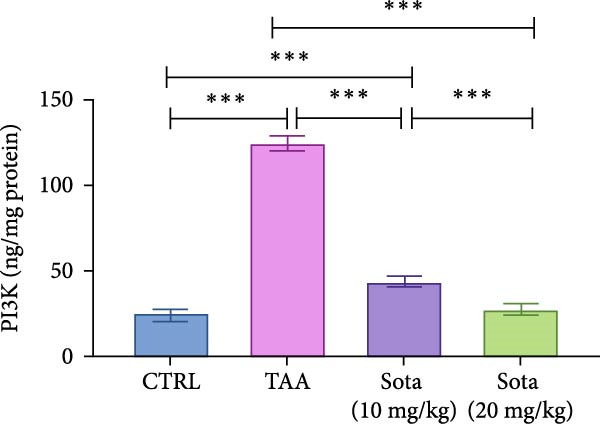
(A)

**Figure:**
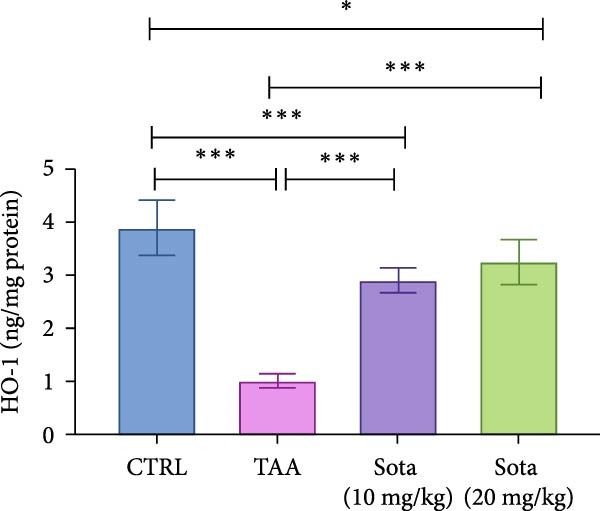
(B)

### 3.7. Effect of Sotagliflozin on Nrf2 Expression in TAA‐Induced Liver Fibrosis

TAA decreased both Nrf2 protein and gene expression by 74.5% and 51.6%, respectively, compared to the control group. Sotagliflozin treatment showed dose‐dependent improvement, with the 20 mg/kg dose increasing protein and gene expression by 234.2% and 108.5%, respectively, compared to the TAA group. The high dose effectively normalized Nrf2 levels, reaching 85.1% and 101% of control values, respectively, as shown in (Figure 7A, B).

Figure 7Sotagliflozin’s effect on Nrf2 gene expression and level in TAA intoxicated rats. (A) Nrf2 gene expression and (B) Nrf2 protein expression. Data are expressed as means ± SD. Significant difference is considered at *p* < 0.05. ∗ vs. in corresponding pairwise at *p* < 0.05, ∗∗ vs. in corresponding pairwise at *p* < 0.01, ∗∗∗ vs. corresponding pairwise at *p* < 0.001. TAA: thioacetamide; Sota: sotagliflozin; Nrf2: nuclear factor erythroid 2‐related factor 2.

**Figure:**
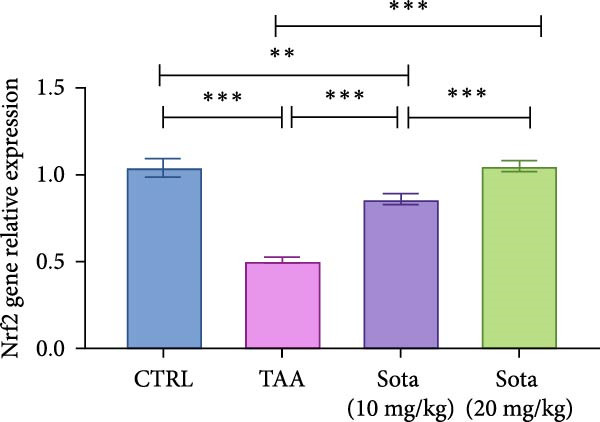
(A)

**Figure:**
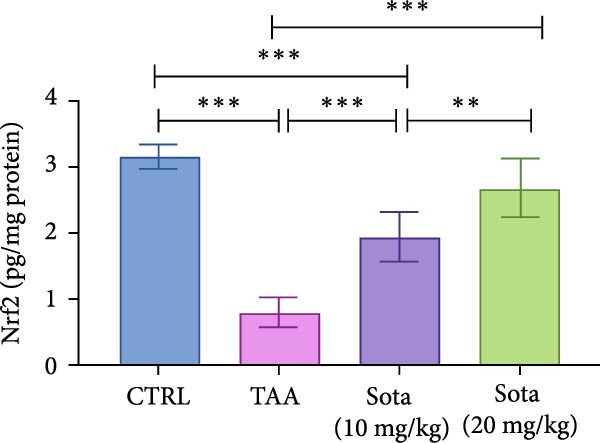
(B)

### 3.8. Effect of Sotagliflozin on SIRT1 Expression in TAA‐Induced Liver Fibrosis

TAA significantly decreased both SIRT1 protein and gene expression by 71% and 81.2%, respectively, compared to the control group. Sotagliflozin treatment showed dose‐dependent improvement, with the 20 mg/kg dose increasing protein and gene expression by 200.9% and 356.2%, respectively, compared to the TAA group. The high dose effectively normalized SIRT1 levels, reaching 87.2% and 85.6% of the control values, respectively (Figure 8A, B).

Figure 8Sotagliflozin’s effect on Sirt1 gene expression and level in TAA intoxicated rats. (A) Sirt1 gene expression and (B) Sirt1 protein expression. Data are expressed as means ± SD. Significant difference is considered at *p* < 0.05. ∗ vs. in corresponding pairwise at *p* < 0.05, ∗∗ vs. in corresponding pairwise at *p* < 0.01, ∗∗∗ vs. corresponding pairwise at *p* < 0.001. TAA: thioacetamide; Sota: sotagliflozin; SIRT1: sirtuin‐1.

**Figure:**
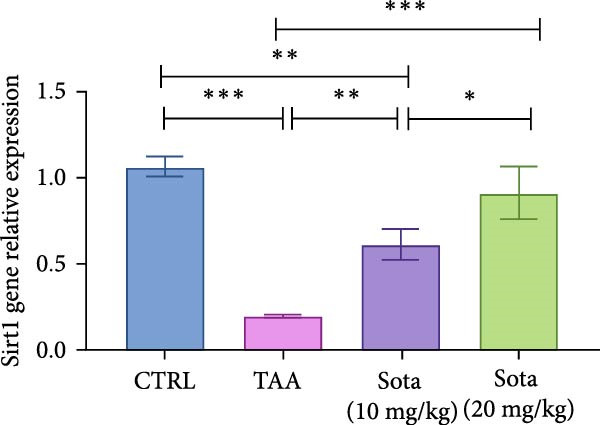
(A)

**Figure:**
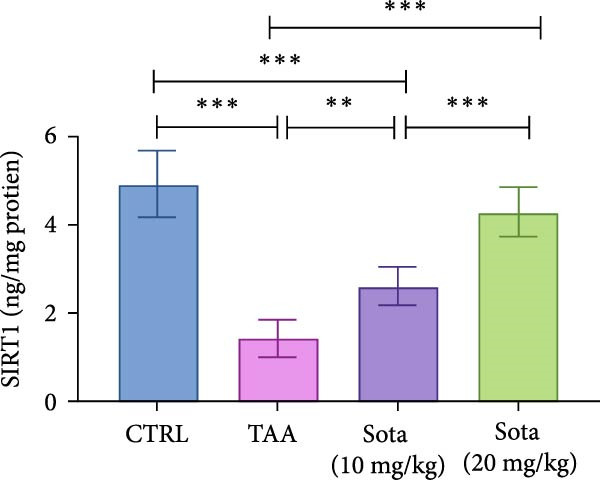
(B)

### 3.9. Histopathological Analysis of Liver Tissue

**Figure 9:**
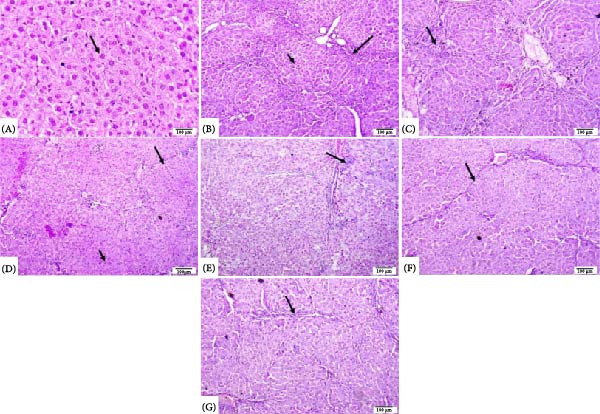
Photomicrograph of rat liver (H&E 100 µm). (A) Control group showing normal histological structure of hepatocytes (arrow). (B) TAA group showing bridging fibrosis and pseudolobulation (long arrow) and necrosis and degeneration of hepatocytes (short arrow). (C) TAA group exhibiting portal area fibroplasia and infiltration of mononuclear inflammatory cells (arrow). (D) TAA + Sota 10 mg/kg showing moderate interlobular fibrosis (long arrow) and degeneration and necrosis of some hepatocytes (short arrow). (E) TAA + Sota 10 mg/kg showing portal areas mild fibrosis and mild inflammatory cells infiltration (arrow). (F) TAA + Sota 20 mg/kg showing mild bridging fibrosis with normal hepatocytes (arrow). (G) TAA + Sota 20 mg/kg showing portal area mild fibrosis (arrow).

Concerning the control group, this group exhibited a normal histological structure of the liver Figure 9A. The TAA group exhibited bridging fibrosis and pseudolobulation. Additionally, there was necrosis and degeneration of hepatocytes (Figure 9B), and the portal area exhibited severe fibroplasia and infiltration of mononuclear inflammatory cells (Figure 9C). TAA + Sota 10 mg/kg showed mild to moderate interlobular fibrosis with degeneration and necrosis of some hepatocytes (Figure 9D) and portal areas showed mild fibrosis, and mild inflammatory cell infiltration (Figure 9E). TAA + Sota 20 mg/kg showed mild bridging fibrosis with normal hepatocytes (Figure 9F), and the portal area showed mild fibrosis (Figure 9G).

### 3.10. The score for histopathological liver lesions

The severity of the recorded liver lesions was assigned a score, as indicated in Table 2.

**Table 2:** Liver histopathological grading in treated groups.

Lesions	Control group	TAA group	TAA + Sota 10 mg/kg	TAA + Sota 20 mg/kg
Bridging fibrosis and pseudolobulation	0	3	1	1
Necrosis of hepatocytes	0	2	1	0
Degeneration of hepatocytes	0	2	1	0
Portal fibroplasia	0	3	1	1
Infiltration of inflammatory cells in portal areas	0	3	1	0

All animals in the group (*n* = 5) had a score of 0 if they had no lesion, 1 if they had a score of less than 30%, 2 if they had a score between 30% and 50%, and 3 if they had a score of greater than 50%.

### 3.11. Immunostaining Findings of Nrf2 and p‐AKT in Rat Liver

**Figure 10:**
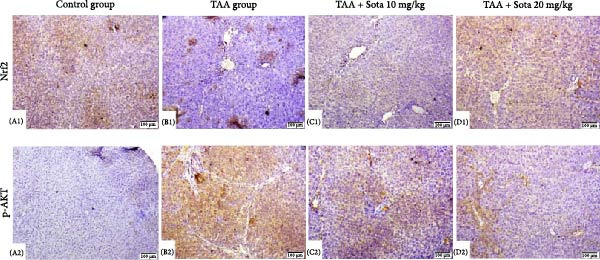
Immunostaining of Nrf2 and p‐AKT in rat liver. (A1, A2) Control group displaying robust expression of Nrf2 and very weak immune expression of p‐AKT. (B1, B2) The TAA group showed weak immunoreactivity of Nrf2 and strong immunoreactivity of p‐AKT. (C1, C2) TAA + Sota 10 mg/kg showing moderate expression of Nrf2 and p‐AKT. (D1, D2) TAA + Sota 20 mg/kg demonstrating robust Nrf2 expression and weak immune‐reactive cells of p‐AKT (Nrf2 and p‐AKT 100 µm).

Immunostaining expression of Nrf2 and p‐AKT % area in liver tissue was illustrated in Figure 10. The control group displayed robust expression of Nrf2 and very weak immune expression of p‐AKT (Figure 10A). The TAA group showed weak immunoreactivity for Nrf2, whereas p‐AKT revealed strong immunoreactivity (Figure 10B). TAA + Sota 10 mg/kg showed an upsurge in the expression of Nrf2 and a significant reduction in the immune‐staining reaction of p‐AKT (Figure 10C). TAA + Sota 20 mg/kg showed a restoration of strong Nrf2 expression. Additionally, this group exhibited weakly reactive immune cells of p‐AKT (Figure 10D). Figure 11 displayed the liver Nrf2 (Figure 11A) and p‐AKT (Figure 11B) immunostaining expression area percentages.

Figure 11Immunostaining expression area % of (A) Nrf2 and (B) p‐AKT in the liver. Data are expressed as means ± SD. Significant difference is considered at *p* < 0.05. ∗ vs. in corresponding pairwise at *p* < 0.05, ∗∗ vs. in corresponding pairwise at *p* < 0.01, ∗∗∗ vs. corresponding pairwise at *p* < 0.001. TAA: thioacetamide; Sota: sotagliflozin; Nrf2: nuclear factor erythroid 2‐related factor 2; p‐AKT: phosphorylated protein kinase B.

**Figure:**
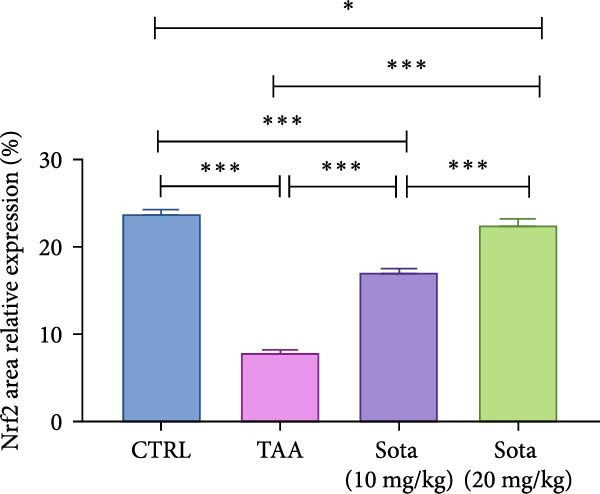
(A)

**Figure:**
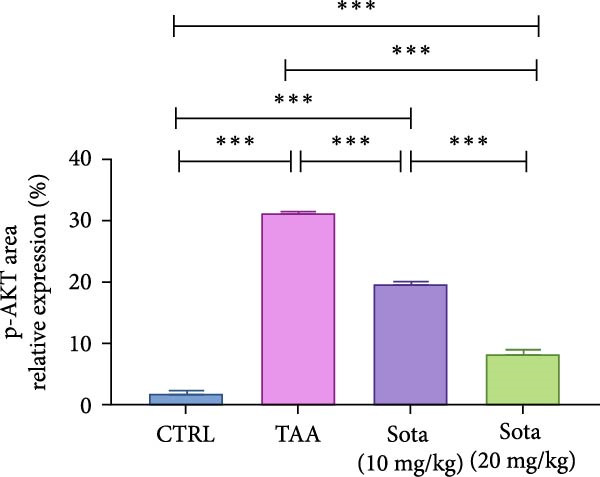
(B)

## 4. Discussion

Liver cirrhosis progressively damages the liver, leading to ECM accumulation and eventual organ failure [34, 35]. SGLT2 inhibitors, originally antidiabetic agents, improve insulin sensitivity by reducing hepatic glucose output [36–38]. In cystic fibrosis models, sotagliflozin reduced portal and lobular inflammation and fibrosis, supporting its potential hepatoprotective effects [20].

The organosulfur compound TAA (C_2_H_5_NS) is commonly used to create animal models of acute and chronic liver injury that closely mimic human liver fibrosis [2]. When CYP450 converts TAA, it forms “TAA‐sulfur dioxide (TAA‐S‐dioxide),” a dangerous reactive metabolite that adheres to liver macromolecules and parenchyma to trigger necrosis and activate HSCs [39, 40]. Because of the breakdown of TAA S‐dioxide, cellular membranes become weaker and more porous, making it easier for the liver enzymes AST and ALT to escape [24, 41]. Our investigation found that TAA negatively affected liver cells, as liver enzymes increased and albumin levels plummeted. These findings matched earlier research [33, 42, 43]. Sotagliflozin lowered liver enzymes and increased albumin. Early research by Liang et al. [20] verified these findings [20]. Eight meta‐analyses found that SGLT2 inhibitors reduced ALT and AST [44].

Liver fibrosis might worsen hepatic damage due to lipid metabolism disturbances. We found that TAA treatment altered lipid homeostasis, resulting in elevated blood TAG and TC [45]. Despite these changes, sotagliflozin dramatically lowered TAG and cholesterol. Sotagliflozin lowers lipids, suggesting it enhances hepatic lipid metabolism pathways by increasing insulin sensitivity and fat consumption [46, 47].

Furthermore, the pathophysiology of liver fibrosis is significantly influenced by oxidative stress [48, 49]. Our study demonstrates that TAA intoxication leads to significant increases in oxidative stress markers such as “MDA” and decreases in antioxidant capacity, as evidenced by declined GSH levels and SOD activity. These findings concur with earlier research [50, 51]. Treatment with sotagliflozin effectively restored these markers, enhancing the anti‐oxidative defense mechanism crucial for protecting cells against oxidative damage [52]. This response is likely mediated through the activation of the Nrf2 pathway, a key regulator of cellular defense against oxidative stress [47, 52, 53]. Typically, “cytoplasmic Nrf2” and “Kelch‐like ECH‐associated protein 1 (Keap1)” combine to form a complex. The protein Keap1 breaks away from Nrf2 in response to oxidative stress and moves to the nucleus, where it interacts with “antioxidant response elements (AREs)” to start the transcription of genes, including HO‐1 [54]. This study found that while Nrf2 expression and level decreased in the TAA group, it was higher in the groups receiving sotagliflozin therapy. The present result consolidates earlier results that SGLT2 inhibition increases Nrf2 activation and potentiates its antioxidant effects [55].

The SIRT1/Nrf2 signaling pathway is the principal antioxidant effector in the cell. TAA markedly decreased SIRT1 expression and level, a finding confirmed in a previous study [56]. It was shown that SIRT1 expression is significantly upregulated following treatment with sotagliflozin in a dose‐dependent manner. Furthermore, SIRT1 has been shown to activate Nrf2, which counteracts growing oxidative stress by inducing the expression of HO‐1 [48, 57–59]. This leads to the inhibition of the cell apoptosis inflammatory pathway through various inflammation and apoptotic proteins such as TNF‐α, Bax, and Bcl‐2 [60]. In response to oxidation damage, SIRT1 decreases cytokines that promote inflammation and alters the Nrf2/nuclear factor kappa B (NF‐κB) crosstalk. SIRT1 signaling also regulates inflammatory transcription factors, including NF‐κB, which is the primary regulator of several proinflammatory cytokines [61, 62]. Thus, the triggering of the SIRT1/Nrf2 pathway may be responsible for the anti‐oxidative and anti‐inflammatory properties of sotagliflozin treatment.

Sotagliflozin also significantly altered apoptosis and inflammation, which are key factors in liver fibrosis. Additionally, it reduced apoptotic markers, such as BAX and Caspase‐3, and increased BCL‐2, while also lowering proinflammatory cytokines, including IL‐6 and TNF‐α. These effects demonstrate that sotagliflozin can prevent hepatocyte death and inhibit the progression of fibrogenesis. Liang et al. [20] study supports our findings demonstrating the anti‐inflammatory actions of sotagliflozin, as it was shown that the use of sotagliflozin resulted in a reduction in NF‐κB levels, a notable increase in lobular and portal inflammation, and a reduction in proinflammatory cytokines such as TNF‐α and IL‐6 in the liver of the cystic fibrosis animal model. Other reports of SGLT inhibitors in cardiovascular and renal disease also support this action of sotagliflozin [63, 64]. The involvement of inflammatory mediators, BAX, Caspase‐3, BCL2, and SIRT1/Nrf2 pathways in the hepatoprotective effects of medications has been reported before in different scenarios [35]. However, other agents target this signaling pathway directly or indirectly, such as quercetin [65], salvianolic acid A [66], captopril [67], and chitosan [68].

In parallel to apoptosis, cell survival, and proliferation are influenced by the phosphatidylinositol 3 kinase/protein kinase B (PI3K/Akt) pathway [23, 33, 39, 69]. AKT, a downstream of PI3K, controls several cellular processes [70]. Hepatic fibrogenesis is documented to be influenced by the PI3K/Akt pathway [71, 72]. The PI3K/AKT signaling pathway is intimately linked to both HSC activation and ECM production [73]. Inhibiting the PI3K/AKT signal pathway can effectively prevent liver damage, enhance liver function, and lower the generation and deposition of collagen [74, 75]. Through the control of central inflammatory cytokines, PI3K, and Akt also contribute to the activation of innate immune cells [76]. Hepatic disorders frequently exhibit a constitutive molecular alteration, including the loss of regulation of the PI3K/AKT pathway in hepatocytes [77]. As a result, one of the major pharmacological approaches for managing liver fibrosis is the PI3K/AKT pathway [78, 79]. Sotagliflozin strongly inhibits the PI3K/AKT pathway [80], thereby counteracting the TAA‐induced activation of the PI3K/AKT pathway [81]. From these results, it can be concluded that sotagliflozin may help in the treatment of hepatic fibrosis by blocking the PI3K/AKT signaling pathway.

Although expression data strongly suggest dual modulation of SIRT1/Nrf2 and PI3K/AKT pathways, pharmacological inhibition, or gene‐silencing approaches were not performed. This remains a limitation, and future studies are warranted to validate causality. The TAA‐induced model reflects chemically driven fibrosis but may not fully replicate the heterogeneity of human liver disease. Moreover, prolonged sotagliflozin therapy may entail risks such as dehydration or ketoacidosis, particularly in advanced chronic liver disease. These limitations should be taken into account when translating the findings into clinical settings.

## 5. Conclusion

The effect of sotagliflozin in liver fibrosis is controlled by targeting the metabolic, oxidative, and inflammatory responses. SIRT1/Nrf2 and PI3K/AKT signaling pathways are two of the key network pathways in which sotagliflozin exhibits additional therapeutic benefits when used to modify the pathophysiology of liver fibrosis. Such a multipart therapeutic approach not only enhances liver functionality but also provides a beneficial hepatoprotective effect, thus promising further investigation in clinical trials to realize its potential in protecting and treating liver diseases fully.

## Ethics Statement

This declaration acknowledges that this article adheres to the principles for transparent reporting and scientific rigour of preclinical research as stated in the BJP guidelines for Design and Analysis, and Animal Experimentation, and as recommended by funding agencies, publishers, and other organizations engaged with supporting research.

## Conflicts of Interest

The authors declare no conflicts of interest.

## Author Contributions

**Hossein M. Elbadawy**, **Mohannad A. Almikhlafi, and Mohammed H. Alsubhi**: conceptualization, supervision, validation, visualization, writing – review and editing. **Aya A. Shokry**: investigation, supervision, validation, visualization, writing – review and editing. **Hany M. Fayed**: conceptualization, data curation, investigation, methodology, supervision, validation, visualization, writing – original draft, writing – review and editing. **Bassim M. S. A. Mohamed**: conceptualization, data curation, investigation, resources, writing – original draft, writing – review and editing. **Tuba Esatbeyoglu**: review and editing, funding acquisition. **Sherif M. Afifi**: visualization, writing – review and editing. **Reda M. S. Korany**: methodology, writing – original draft, writing – review and editing. **Marawan A. Elbaset**: conceptualization, data curation, formal analysis, investigation, methodology, software, validation, visualization, writing – original draft, writing – review and editing.

## Funding

The publication of this article was funded by the Open Access Fund of Leibniz Universität Hannover. The authors appreciate Taibah University, Madinah, Kingdom of Saudi Arabia for the research Grant 447‐13‐1028 to support this work. Open Access funding enabled and organized by Projekt DEAL.

## Data Availability

The data supporting the findings of this study are available from Marawan A. Elbaset upon reasonable request.
